# Role of Iodine Status and Lifestyle Behaviors on Goiter among Children and Adolescents: A Cross-Sectional Study in Zhejiang Province, China

**DOI:** 10.3390/nu16172910

**Published:** 2024-08-31

**Authors:** Guangming Mao, Changyuan Zhou, Lichun Huang, Zhe Mo, Danting Su, Simeng Gu, Fanjia Guo, Yuanyang Wang, Zhijian Chen, Ronghua Zhang, Xiaoming Lou, Xiaofeng Wang, Jie Hu, Fang Gu, Bin Dong

**Affiliations:** 1 Department of Environmental Health, Zhejiang Provincial Center for Disease Control and Prevention, 3399 Binsheng Road, Hangzhou 310051, China; gmmao@cdc.zj.cn (G.M.); zhmo@cdc.zj.cn (Z.M.); smgu@cdc.zj.cn (S.G.); fjguo@cdc.zj.cn (F.G.); yywang@cdc.zj.cn (Y.W.); zhjchen@cdc.zj.cn (Z.C.); xmlou@cdc.zj.cn (X.L.); xfwang@cdc.zj.cn (X.W.); 2 Institute of Child and Adolescent Health, School of Public Health, Peking University Health Science Center, No. 38 Xueyuan Road, Haidian District, Beijing 100191, China; zcy1412@stu.pku.edu.cn; 3 Institute of Nutrition and Food Safety, Zhejiang Provincial Center for Disease Control and Prevention, 3399 Binsheng Road, Hangzhou 310051, China; lchhuang@cdc.zj.cn (L.H.); dtsu@cdc.zj.cn (D.S.); rhzhang@cdc.zj.cn (R.Z.); 4 Menzies Health Institute Queensland, Griffith University, Nathan, QC 4111, Australia; jie.hu@griffith.edu.au

**Keywords:** urinary iodine concentration, iodine status, lifestyle behaviors, goiter, child, adolescent

## Abstract

Background: Iodine deficiency is a well-established cause of goiter, while the impact of lifestyle factors on goiter development remains underexplored. The study aims to explore the associations between iodine status, lifestyle factors, and the prevalence of goiter among children and adolescents in Zhejiang Province, China. Methods: A cross-sectional survey was conducted in 2022 using a stratified multistage sampling, involving 2261 children aged 6–17. Among these 1562 participants underwent both urinalysis and thyroid ultrasound. Lifestyle factors were assessed through self-reported questionnaires. Results: The prevalence of goiter in the study population was 10.8%. A high urinary iodine concentration (UIC) (>300 μg/L) was significantly associated with a decreased risk of goiter (OR = 0.49, 95%CI: 0.27–0.88). Excessive recreational screen time and a high frequency of dining out were associated with an increased Tvol, while adequate physical activity and sleep were inversely associated with goiter risk, while the combined effect of high UIC and healthy lifestyle showed a protective effect against goiter. Conclusion: Ensuring adequate iodine status and promoting healthy lifestyles are crucial for preventing goiter and enhancing thyroid health in children and adolescents, suggesting that public health strategies should integrate nutritional and lifestyle interventions.

## 1. Introduction

Goiter is a significant health concern due to its potential adverse health effects. Characterized by an enlarged thyroid gland, goiter is considered to be a common indication of acquired thyroid disease [1] and is linked to a higher risk of overt hypothyroidism [2], particularly in vulnerable populations like children and adolescents due to their increased nutritional requirements during growth spurts [3]. Recent studies indicate that the prevalence of goiter was 3.5% in Chinese school-aged children [4], suggesting that although it is not highly prevalent, it still affects a significant number of individuals and warrants attention. However, existing studies have paid much less attention to lifestyle factors related to goiter compared to iodine status, despite evidence that lifestyle factors can predict other endocrine diseases [5].

Multiple risk factors contribute to the development of goiter. Iodine deficiency is widely recognized as a common cause of goiter, as iodine is essential for thyroid hormone synthesis [6]. Public health initiatives, such as salt iodization programs, have significantly reduced the incidence of iodine deficiency disorders [7]. However, in areas with high water iodine concentration (WIC), excessive iodine is also associated with a higher goiter rate among Chinese school-aged children [8,9]. Monitoring iodine status remains crucial to prevent both deficiency and excess in children and adolescents [10]. Accumulating evidence links sociodemographic factors, such as age, body mass index (BMI), socio-economic status, and area, with goiter [11,12,13,14]. Furthermore, building on previous research on other thyroid disorders, lifestyle factors are potential contributors to the development of goiter [15,16,17]. However, the impact of lifestyle factors on goiter has not been thoroughly investigated, and no study has comprehensively explored the combined effects of iodine nutrition, sociodemographic characteristics, and lifestyle factors in children and adolescents.

Consequently, this cross-sectional study aims to examine the associations between urinary iodine concentration (UIC), lifestyle factors, and goiter among school-aged children and adolescents in Zhejiang Province, China. Additionally, it seeks to elucidate the interplay between various risk factors for goiter, with the goal of providing valuable insights for the development of targeted interventions and public health policies aimed at reducing the burden of thyroid disorders in this population.

## 2. Materials and Methods

### 2.1. Sampling and Participants

This study conducted a cross-sectional survey on a representative sample of children and adolescents in Zhejiang Province in 2022. A stratified multistage sampling technique was performed. First, 16 districts or counties were selected randomly from Zhejiang Province as surveillance sites. Second, for each selected site, three towns or streets were randomly selected. Third, for each selected town or street, two villages or residential committees were randomly selected. Finally, a total of 2261 children and adolescents aged 6–17 in Zhejiang participated in this study. The final number of analyses included 1562 subjects, since all of them underwent both urinalysis and thyroid ultrasound.

The inclusion criteria for the study were children and adolescents aged from 6 to 17 years old at enrollment who were residents and outsiders who lived in the area for more than three years. Children or adolescents who received contrast-enhanced ultrasonography with an iodine-containing contrast agent, or who had taken ethylamiodarone within the past three months, were excluded from the study.

Ethical approval was obtained from the Ethical Committee of the Zhejiang Provincial Chinese Centers for Disease Control and Prevention (CDC) (2022-018-01). Written informed consent was obtained from all participants.

### 2.2. Procedures

#### 2.2.1. Sociodemographic Characteristic

Each enrolled participant completed self-reported questionnaires through interviews with well-trained investigators. Gender, date of birth, and household income per capita in 2022 were recorded. Height and weight were measured by professional public health doctors. BMI was calculated by dividing weight (kg) by the square of height (m^2^). In accordance with the Chinese national screening standard for underweight, overweight, and obesity (WS/T 586-2018, WS/T 456-2014) [18,19], participants were categorized into three groups based on their BMI.

#### 2.2.2. UIC

A 6 mL random spot urine sample was obtained from each participant between July 2022 and November 2022. UIC was measured using the As^3+^-Ce^4+^ catalytic spectrophotometric method [20] (WS/T 107-2006). All iodine laboratories participated in internal quality control and external quality assurance programs run by the CDC. Iodine nutrition was assessed according to the recommended iodine nutrition status evaluation criteria of the WHO [21] (Insufficient: <100 μg/L; Adequate: 100–199 μg/L; Above requirements: 200–299 μg/L; Excessive: ≥300 μg/L).

#### 2.2.3. Lifestyle Factors

Lifestyle factors included moderate- to vigorous-intensity physical activity, recreational screen exposure time, sleep duration, and frequency of dining out. Recreational screen exposure includes activities such as watching television, playing video games, and using electronic devices for recreation and relaxation.

Lifestyle factors were assessed using a self-designed questionnaire, developed following consultations with several public health experts. The questionnaire included items on participants’ average daily time spent in moderate- to vigorous-intensity physical activity, recreational screen exposure, nighttime sleep duration, and the frequency of dining out per week. Recreational screen exposure encompassed activities such as watching television, using a smartphone, playing video games, and using devices like iPods, iPads, or other tablets and computers.

Participants were categorized into two groups for physical activity (<1 h/d and ≥1 h/d) and recreational screen exposure (<2 h/d and ≥2 h/d), respectively. These classifications were based on the WHO Guidelines on Physical Activity and Sedentary Behavior [22], as well as the current global status of screen exposure among adolescents [23]. For sleep duration, the National Sleep Foundation recommends at least 9 h per night for 6–13 years and at least 8 h per night for 14–17 years [24]. Sleep duration was dichotomized as adequate or inadequate based on the recommendations. The frequency of dining out (times/week) was divided into two groups: low frequency (≤2) and high frequency (>2). Participants with missing data on any of the four items were excluded from the lifestyle score calculation to maintain the integrity of the score.

To assess the association between a comprehensive healthy lifestyle and the main outcome, we assigned scores to each criterion as follows: ≥1 h/day of physical activity was assigned 1 point, <2 h/day of recreational screen time was assigned 1 point, sufficient sleep was assigned 1 point, and dining out ≤2 times per week was assigned 1 point. If the criteria were not met, 0 points were assigned. The total lifestyle score, ranging from 0 to 4, was calculated by summing the scores of these four items and categorized into low adherence to a healthy lifestyle (score < 2) and high adherence (score ≥ 2) groups.

#### 2.2.4. Main Outcome

The main outcome of this study is thyroid volume (Tvol) and goiter. The measurements of the maximum length, width, and thickness of both lobes of the thyroid gland were performed using a SonoSite MicroMaxx portable color Doppler ultrasonography device (7.5 MHz transducer, SonoSite Inc., Bothell, WA, USA). The volume of each thyroid lobe was calculated using the formula V(mL) = 0.479 × width(mm) × length(mm) × thickness(mm)/1000. The sum of the volumes of both lobes was the total Tvol. Goiter was defined by age-specific Tvol according to Chinese national diagnostic criteria [25] (WS 276-2007). The participant was diagnosed with goiter if the participant’s Tvol exceeded the corresponding value.

### 2.3. Statistical Analysis

R statistical software (version 4.3.2, R Core Team, Vienna, Australia) was utilized for all analyses [26]. The Shapiro–Wilk test was used to test for normality. Counted data were expressed as numbers and percentages (%). *T*-tests and chi-square tests were applied to test the difference between boys and girls. All tests were two-sided, and *p*-values < 0.05 were considered significant.

Linear regression models and logistic regression models were used to estimate the association between UIC and lifestyle factors (diet, exercise, sleep, and screen exposure) with Tvol and goiter. Firstly, UIC and the four lifestyle factors were separately included in the regression models. Then, based on different UIC levels, lifestyle scores were included in the subgroup regression models as continuous and counted variables. Firstly, we ran a model without adjusting for any factors (model 1). The basic statistical model 1 was not adjusted for any factors. Demographic characteristics, including gender (boy/girl), age (year, continuously), and household income per capita (yuan, continuously), were adjusted in model 2. In model 3, the BMI group (overweight or obesity/normal weight/underweight) was included as a covariate in addition to those covariates in model 2. The results of the linear regression model are expressed as coefficients and standard errors, while the results of the logistic regression model are expressed as odds ratios (OR) and 95% confidence intervals (95%CI).

## 3. Results

### 3.1. Participants Characteristics

This study included 1562 participants, with 799 boys and 763 girls. The mean age was 11.2 ± 3.3 years. The distribution of the sample by sex and age is depicted in the population pyramid shown in Figure S1 in the Supplementary Materials. Participants were divided into three age groups: 6–9 years (35.6%), 10–13 years (34.7%), and 14–17 years (29.7%). The mean BMI was 18.38 kg/m^2^, with 65.6% being normal weight and 24.0% being overweight or obese. Participants engaged in an average of 0.38 h of physical activity per day, with only 11.2% meeting the recommended ≥1 h of daily physical activity. Recreational screen exposure averaged 1.48 h per day, with 26.9% limiting their screen time to less than 2 h per day. The mean sleep duration was 8.91 h per day, with 76.0% achieving adequate sleep duration. On average, participants dined out 2.12 times per week, with 78.2% dining out two times or fewer per week. The mean UIC was 217.09 μg/L, with 16.1% having UIC < 100 μg/L, 39.8% between 100 and 199 μg/L, 25.1% between 200 and 299 μg/L, and 19.0% ≥ 300 μg/L. The median thyroid volume (Tvol) was 4.14 mL, and the prevalence of goiter was 10.8% (Table 1). Figures S2–S4 illustrate the distribution of UIC status, goiter status, and adherence to a healthy lifestyle across various gender and age groups, respectively, providing a comprehensive overview of these factors within the study population.

### 3.2. Association between UIC and Outcomes

Table 2 presents the results of linear and logistic models regarding the association between UIC (continuous/count) and the main outcome of this study (Tvol and goiter). There was no significant association between UIC and Tvol (*p* > 0.05), but subjects with a higher UIC had a low risk of goiter (*p* = 0.022). Specifically, compared to those with UIC under 100 μg/L, participants with high UIC (≥300 μg/L) showed a significantly lower risk of goiter after adjusting for potential confounding factors (OR = 0.49, 95%CI, 0.27–0.88).

**Figure 1:**
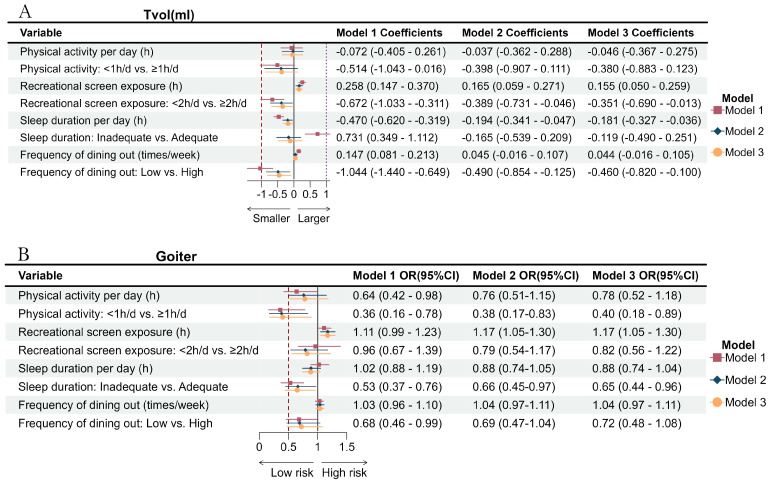
Effect of physical activity, recreational screen exposure, sleep duration, and frequency of dining out on Tvol (**A**) and goiter (**B**). Model 1 did not adjust for any factors. Model 2 adjusted for gender, age, and household income per capita. Model 3 additionally adjusted for the BMI group.

Results of regression models regarding the association between four lifestyle factors (continuous data and count data) and Tvol and goiter are shown in Figure 1. Tvol was positively associated with recreational screen exposure and high frequency of dining out, while it was inversely associated with sleep duration in three models. Longer screen exposure and higher frequency of dining out are associated with larger Tvol, while longer sleep duration is associated with a decreased Tvol in three models. Longer physical activity (OR = 0.36, 95%CI, 0.16–0.78; OR = 0.38, 95%CI, 0.17–0.83; OR = 0.40, 95%CI, 0.18–0.89, respectively) and sleep duration (OR = 0.53, 95%CI, 0.37–0.76; OR = 0.66, 95%CI, 0.45–0.97; OR = 0.65, 95%CI, 0.44–0.96, respectively) is associated with lower risk of goiter. The odds of goiter were 1.17 higher for each additional hour per day of recreational screen time in model 2 and 3, while no association between limited recreational screen exposure (<2h/d) and goiter was identified in all models (*p* > 0.05).

**Figure 2:**
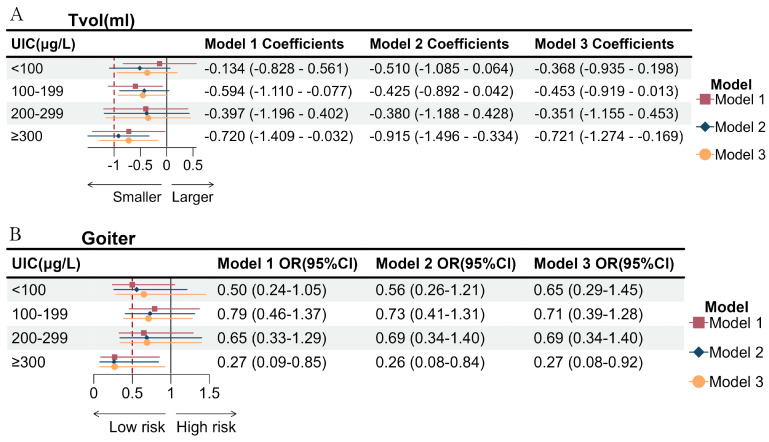
Effect of comprehensive lifestyle (count) on Tvol (**A**) and goiter (**B**) in different iodine status. Model 1 did not adjust for any factors. Model 2 adjusted for gender, age, and household income per capita. Model 3 additionally adjusted for the BMI group.

Furthermore, Table 3 and Figure 2 show the associations between lifestyle scores and the main outcome in subgroups classified by the level of UIC. For participants with UIC varying from 100 μg/L to 199 μg/L and high UIC, a healthy lifestyle had an inverse association with Tvol in three models. Moreover, subjects with low UIC (<100 μg/L) (OR = 0.62, 95%CI, 0.40–0.95; OR = 0.57, 95%CI, 0.36–0.91, respectively) and high UIC (OR = 0.28, 95%CI, 0.15–0.54; OR = 0.29, 95%CI, 0.15–0.55, respectively) with healthier lifestyles had a decreased risk of goiter in model 1 and 2. Similar associations were observed in high-UIC groups in model 3 (OR = 0.28, 95%CI, 0.14–0.56).

In Figure 2, individuals with a high UIC and a low-risk lifestyle had a smaller Tvol and a decreased risk of goiter in three models (OR = 0.27, 95%CI, 0.09–0.85; OR = 0.26, 95%CI, 0.08–0.84; OR = 0.27, 95%CI, 0.08–0.92, respectively).

A further subgroup analysis was employed to examine the consistency of the associations in three different age groups, finding that children aged over 10 years old with healthier lifestyles had a decreased risk of elevated Tvol and goiter (see Tables S1 and S2 in the Supplementary Materials).

## 4. Discussion

In this study, a significant association was observed between iodine status and goiter among 1562 school-aged children and adolescents in Zhejiang Province, China. Our findings reveal that children aged 6–17 with excessive UIC, coupled with a healthy lifestyle, exhibited a lower risk of goiter and smaller Tvol, particularly noticeable in individuals aged over 10 years.

The prevalence of goiter in this study was 10.8%, which is significantly higher than the national average of 3.5% [4] and yet is similar to the prevalence reported in high WIC areas (10.2%) [9]. This discrepancy underscores the potential regional variations in iodine intake and the need for localized public health interventions. The higher prevalence in our sample may be attributed to specific environmental or dietary factors prevalent in Zhejiang Province that warrant further investigation.

Our results indicate that while there was no significant association between UIC and Tvol, participants with high UIC (>300 μg/L) had a significantly decreased risk of goiter. This finding regarding Tvol aligns with previous research conducted on school-aged children in Zhejiang Province and an iodine-sufficient region in Turkey [27,28]. However, in some provinces with high WIC in China, high UIC is associated with a smaller average thyroid volume [29]. This discrepancy may be attributed to different physiological mechanisms. Tvol mainly reflects the growth and proliferation of thyroid cells, whereas goiter formation is often associated with thyroid dysfunction and hormonal regulation. High UIC might prevent goiter formation through mechanisms that do not significantly impact overall Tvol. In addition, regional differences in WIC may also contribute to the discrepancy [30]. Despite some studies finding a significant association between extremely high UIC levels and an increased prevalence of goiter, these studies often define high UIC as levels greater than 500 μg/L or higher [8,31], indicating a need for further research to elucidate this relationship.

Our study shows that longer recreational screen exposure, shorter sleep duration, and higher frequency of dining out are related factors of larger Tvol, while enough physical activity (≥1 h/d), recreational screen time, and adequate sleep are associated with a decreased risk of goiter. Obese and overweight children tend to have larger Tvol and a higher risk of goiter [32]. Mounting evidence indicates the effect of screen exposure and physical activity on children’s BMI status [33]. Inadequate sleep predicts unhealthy eating behaviors in adolescents and children, including higher caloric intake and fewer fruits or vegetables [34,35], which may be attributive to obesity [36]. Children aged 6–17 years old are the main group dining out in China [37], and dining out ≥3 times per week has a significant positive effect on overweight and obesity in this population, particularly boys [38]. Lifestyle behaviors play a crucial role in modulating thyroid health, possibly by modulating BMI in children aged 6–17 years old.

The association between physical activity and thyroid health can be explained by the change in thyroid hormone levels as well. Goiter, the abnormal enlargement of the thyroid gland, emerges due to a compensatory increase in Thyroid-Stimulating Hormone (TSH) release triggered by lower T_4_ levels [6]. In the general population, sustained physical exercise is associated with reduced levels of TSH [39], and increased physical activity can reduce TSH levels in an L-shaped curve [40]. Some research explains the reduction by the negative feedback on hypothalamic–pituitary–thyroid axis caused by higher T_3_ and T_4_ levels [41], while others explain it by the suppression of hypothalamic function due to reduced leptin [42]. The changes in TSH levels may explain the negative association between adequate physical activity and thyroid enlargement [43]. However, research has not been consistent about the effectiveness of physical activity in improving TSH levels in different populations [44], appearing to depend on the intensity and duration of exercise protocol [45]. Further research on school children and adolescents is necessary.

Few studies have explored the association between screen exposure or sedentary behavior and thyroid health. However, prolonged sedentary behavior may regulate thyroid health by impacting overall metabolic health and endocrine function, often accompanied by reduced physical activity [46]. Future research should directly investigate the link between these factors.

Sleep may also modulate thyroid health by influencing endocrine function. Research suggests that TSH levels tend to increase in shift workers, possibly due to inadequate sleep and poor sleep quality [47]. Upon sleep improvement, TSH concentrations typically return to normal levels [48]. However, another study found no significant association between sleep deprivation and TSH levels [49]. Previous investigations have primarily focused on the general population, and future research should explore this relationship further in children and adolescents.

Previous research did not find a significant effect of non-iodized salt on goiter when school-aged children were dining out [50]. Among Canadian adults and US adolescents, a higher frequency of dining out is associated with lower diet quality, including lower vegetable and milk intake [51,52]. Lower milk intake and higher dark green vegetable intake are associated with a higher risk of goiter in Ethiopian children [53]. Future research on Chinese children and adolescents is needed to clarify the mechanism of dining out on the development of goiter.

Based on these four lifestyle factors, our study found that the effect of healthy lifestyle factors on Tvol and goiter varied depending on iodine status. A healthier lifestyle was inversely associated with the risk of goiter, particularly in children with high UIC. Previous research indicated the effect of lifestyle on some other thyroid diseases, such as thyroid dysfunction [16], Graves’ disease [17], and thyroid nodules [54]. Integrating multiple risk factors and their combined effects on goiter, our findings contribute to this growing body of evidence, suggesting that lifestyle modifications may complement iodine intake interventions to improve thyroid health.

The findings of this study highlight the need for integrated public health strategies that address both nutritional and lifestyle factors to prevent goiter among children and adolescents. While ensuring normal iodine intake remains a cornerstone of thyroid health interventions, our study suggests that promoting healthy lifestyle behaviors is equally important. In addition to ensuring normal iodine intake, public health campaigns should focus on encouraging regular physical activity, limiting recreational screen time, promoting sufficient sleep, and decreasing the frequency of dining out among young populations.

Despite the strengths of our study, including a comprehensive assessment of lifestyle factors, there are several limitations to consider. First, the cross-sectional design limits our ability to determine causality. Longitudinal studies are needed to confirm the associations we observed and explore the causal mechanisms underlying these relationships. Second, our reliance on self-reported data for lifestyle factors may introduce recall bias and measurement errors. Participants might overestimate or underestimate their physical activity, screen time, and sleep duration. Future studies should consider using objective measures, such as accelerometers for physical activity and sleep monitors, to obtain more accurate data. Moreover, while we adjusted for several potential confounders, residual confounding cannot be entirely ruled out. Factors such as dietary patterns, genetic predispositions, and environmental exposures may also play a role in thyroid health and should be considered in future research.

## 5. Conclusions

Our findings indicate that children aged 6–17 with higher UIC levels, particularly those with UIC ≥ 300 μg/L, have a reduced risk of developing goiter. This association is more pronounced in older children, highlighting the importance of monitoring iodine intake to prevent thyroid disorders. Furthermore, our study underscores the role of lifestyle behaviors in modulating this relationship. Specifically, individuals with healthier lifestyle habits are less likely to develop goiter, particularly among those with elevated UIC levels. These findings emphasize the need for public health strategies that not only ensure adequate iodine intake, but also promote healthy lifestyle behaviors to support thyroid health. Further research is warranted to explore the underlying mechanisms driving these associations.

## Figures and Tables

**Table 1:** Characteristics of participants.

Variables	*N*		Boys (*N* = 799)	Girls (*N* = 763)	*p*
		Mean (SD)/*N* (%)	Mean (SD)/*N* (%)	Mean (SD)/*N* (%)	
Age (year)	1562	11.19 (3.34)	11.12 (3.26)	11.27 (3.42)	0.393
Age group	1562				0.123
6–9 years		556 (35.6%)	281 (35.2%)	275 (36.0%)	
10–13 years		542 (34.7%)	295 (36.9%)	247 (32.4%)	
14–17 years		464 (29.7%)	223 (27.9%)	241 (31.6%)	
Height (cm)	1562	145.6 (18.5)	147.2 (19.8)	144.0 (16.8)	0.001
Weight (kg)	1562	40.7 (16.6)	42.7 (18.4)	38.6 (14.3)	<0.001
BMI (kg/m^2^)	1562	18.4 (4.1)	18.8 (4.2)	18.0 (3.9)	<0.001
BMI group:	1562				<0.001
Underweight		162 (10.4%)	91 (11.4%)	71 (9.3%)	
Normal weight		1025 (65.6%)	467 (58.4%)	558 (73.1%)	
Overweight/obesity		375 (24.0%)	241 (30.2%)	134 (17.6%)	
Household income per capita	1464	33,844.30 (29,847.81)	33,939.64 (29,605.48)	33,743.05 (30,123.58)	0.900
Physical activity (h/d)	1550	0.38 (0.52)	0.42 (0.57)	0.33 (0.46)	0.001
Adequate physical activity (≥1 h/d)	1550	173 (11.2%)	101 (12.6%)	72 (9.4%)	0.046
Recreational screen exposure (h/d)	1527	1.48 (1.38)	1.57 (1.42)	1.40 (1.34)	0.017
Limited screen exposure (<2 h/d)	1527	410 (26.9%)	235 (29.4%)	175 (22.9%)	0.004
Sleep duration (h/d)	1287	8.91 (1.07)	8.90 (1.07)	8.92 (1.08)	0.756
Adequate sleep duration ^a^	1287	978 (76.0%)	498 (62.3%)	480 (62.9%)	0.960
Frequency of dining out (times/week)	1294	2.12 (2.43)	2.09 (2.36)	2.16 (2.50)	0.598
Low frequency of dining out (≤2)	1294	1012 (78.2%)	519 (78.4%)	493 (78.0%)	0.918
Urinary iodine concentration (μg/L)	1562	217.09 (154.06)	214.78 (148.92)	219.51 (159.33)	0.545
UIC (μg/L)	1562				0.020
<100		252 (16.1%)	120 (15.0%)	132 (17.3%)	
100–199		621 (39.8%)	347 (43.4%)	274 (35.9%)	
200–299		392 (25.1%)	184 (23.0%)	208 (27.3%)	
≥300		297 (19.0%)	148 (18.5%)	149 (19.5%)	
Tvol (mL)	1562	4.75 (2.90)	4.72 (2.94)	4.79 (2.86)	0.628
Goiter	1562	168 (10.8%)	77 (9.6%)	91 (11.9%)	0.168

^a^ The National Sleep Foundation recommends at least 9 h per night for 6–13 year olds and at least 8 h per night for 14–17 year olds, based on which sleep duration was dichotomized as adequate or inadequate.

**Table 2:** Effect of iodine status on Tvol and goiter.

Thyroid-Related Indicators	Variables	Mean (SD)	Model 1 ^a^			Model 2 ^b^			Model 3 ^c^		
			*β*	*SE*	*p*	*β*	*SE*	*p*	*β*	*SE*	*p*
Tvol (mL)	UIC (μg/L)	4.75 (2.90)	0.0005	0.0005	0.264	−0.0001	0.0004	0.806	−0.00005	0.0004	0.909
	UIC (μg/L)										
	<100	4.65 (2.53)	Ref.			Ref.			Ref.		
	100–199	4.80 (2.69)	0.086	0.217	0.691	−0.233	0.198	0.240	−0.222	0.196	0.257
	200–299	4.74 (2.77)	0.159	0.234	0.498	−0.101	0.213	0.636	−0.093	0.211	0.658
	≥300	4.81 (3.43)	0.150	0.249	0.545	−0.367	0.228	0.107	−0.333	0.226	0.140
		***N* (%)**	**OR**	**95%CI**	***p***	**OR**	**95%CI**	***p***	**OR**	**95%CI**	***p***
Goiter	UIC (μg/L)	168 (10.8%)	0.998	0.996–0.999	0.002	0.998	0.997–1.00	0.015	0.998	0.997–1.00	0.022
	UIC (μg/L)										
	<100	40 (15.9%)	Ref.			Ref.					
	100–199	67 (10.8%)	0.64	0.42–0.98	0.039	0.71	0.46–1.11	0.123	0.71	0.45–1.11	0.126
	200–299	41 (10.5%)	0.62	0.39–0.99	0.045	0.73	0.45–1.19	0.202	0.75	0.46–1.22	0.237
	≥300	20 (6.7%)	0.38	0.21–0.67	0.001	0.48	0.26–0.85	0.014	0.49	0.27–0.88	0.018

^a^ Model 1 did not adjust for any factors (*n* = 1562). ^b^ Model 2 adjusted for gender, age, and household income per capita (*n* = 1464). ^c^ Model 3 additionally adjusted for the BMI group (*n* = 1464).

**Table 3:** Effect of comprehensive lifestyle (continues) on Tvol and goiter in different iodine status.

Thyroid-Related Indicators	UIC (μg/L)	Model 1 ^a^			Model 2 ^b^			Model 3 ^c^		
		*β*	*SE*	*p*	*β*	*SE*	*p*	*β*	*SE*	*p*
Tvol (mL)	<100	−0.210	0.217	0.334	−0.370	0.181	0.042	−0.225	0.182	0.216
	100–199	−0.372	0.155	0.017	−0.301	0.139	0.030	−0.307	0.138	0.027
	200–299	−0.250	0.270	0.356	−0.137	0.271	0.614	−0.117	0.270	0.664
	≥300	−0.628	0.217	0.004	−0.694	0.183	<0.001	−0.595	0.173	<0.001
		**OR**	**95%CI**	***p***	**OR**	**95%CI**	***p***	**OR**	**95%CI**	***p***
Goiter	<100	0.62	0.40–0.95	0.027	0.57	0.36–0.91	0.019	0.66	0.40–1.07	0.092
	100–199	0.77	0.57–1.06	0.112	0.78	0.56–1.09	0.152	0.78	0.56–1.09	0.144
	200–299	0.84	0.55–1.30	0.446	0.86	0.55–1.36	0.523	0.87	0.55–1.36	0.535
	≥300	0.28	0.15–0.54	<0.001	0.29	0.15–0.55	<0.001	0.28	0.14–0.56	<0.001

^a^ Model 1 did not adjust for any factors (*n* = 1276). ^b^ Model 2 adjusted for gender, age, and household income per capita (*n* = 1184). ^c^ Model 3 additionally adjusted for BMI group (*n* = 1184).

## Data Availability

The raw data supporting the conclusions of this article will be made available by the authors on request.

## Supplementary Materials

The following supporting information can be downloaded at: https://www.mdpi.com/article/10.3390/nu16172910/s1, Figure S1: Age and Sex Distribution of the Study Sample; Figure S2: UIC status by Gender and Age Groups; Figure S3: Goiter Status by Gender and Age Groups. Figure S4: Adherence to a Healthy Lifestyle by Gender and Age Groups.; Table S1: Effect of lifestyle (continues) on thyroid volume and goiter in different iodine nutritional status and different age groups; Table S2: Effect of lifestyle (count) on thyroid volume and goiter in different iodine nutritional status and different age groups.
