# Supplementary material for: Role of Iodine Status and Lifestyle Behaviors on Goiter among Children and Adolescents: A Cross-Sectional Study in Zhejiang Province, China
Source: Nutrients. 2024 Aug 31;16(17):2910. doi: 10.3390/nu16172910 (PMC11397286; doi:10.3390/nu16172910)
Supplement: Supplementary file 1 [file nutrients-16-02910-s001.zip › nutrients-3159955-supplementary.pdf]

Supplementary material

Role of Iodine Status and Lifestyle Behaviors on Goiter among Children and Adolescents: A Cross-Sectional Study in Zhejiang Province, China

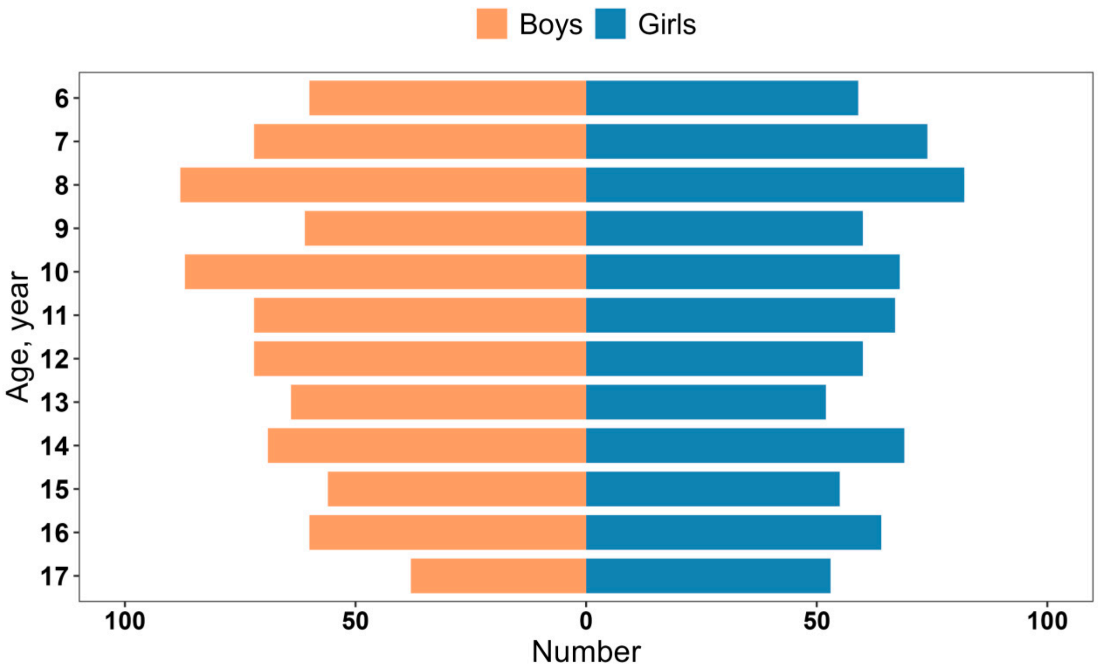

Figure S1. Age and Sex Distribution of the Study Sample

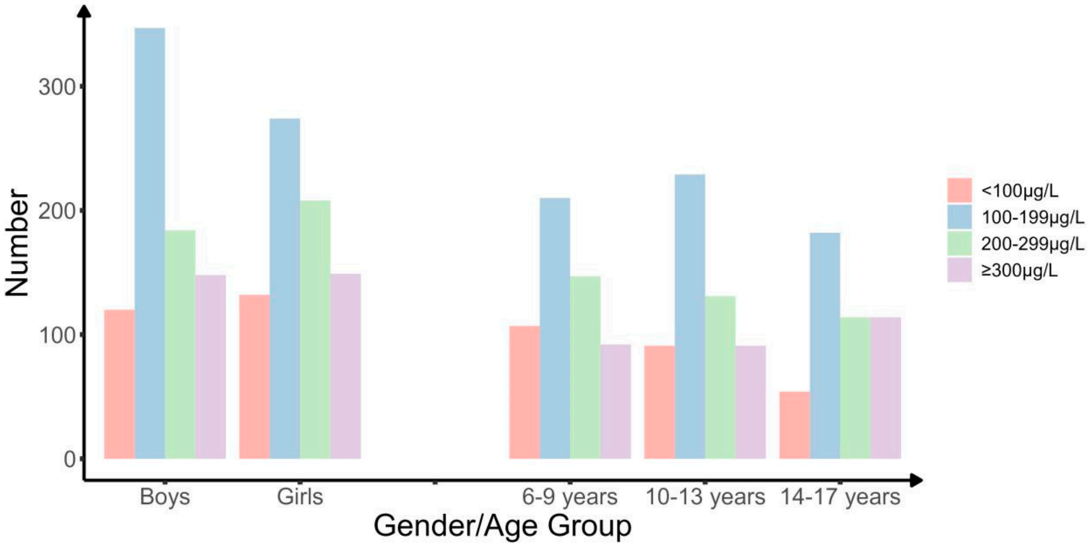

Figure S2. UIC Status by Gender and Age Groups

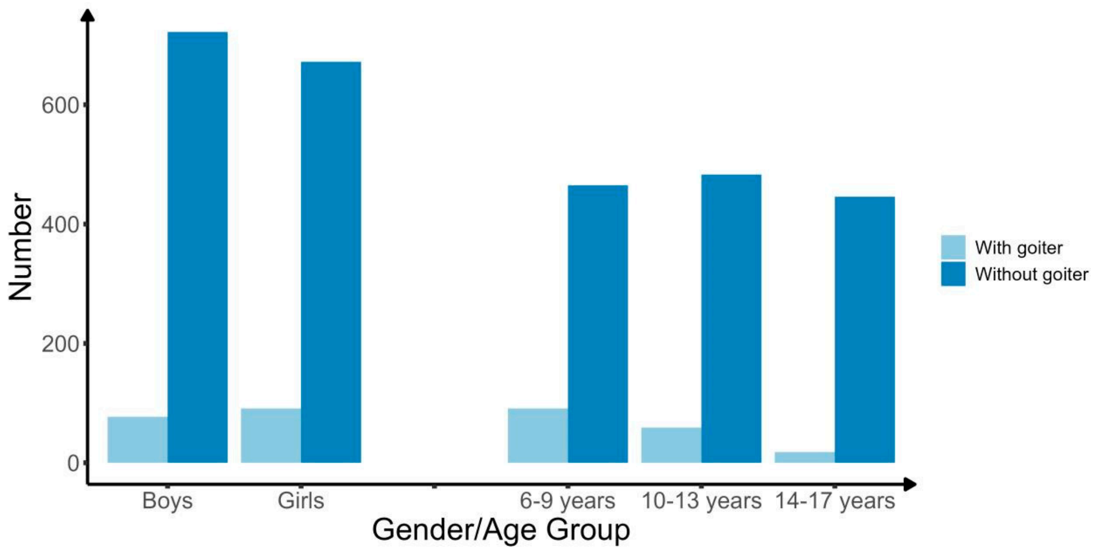

Figure S3. Goiter Status by Gender and Age Groups

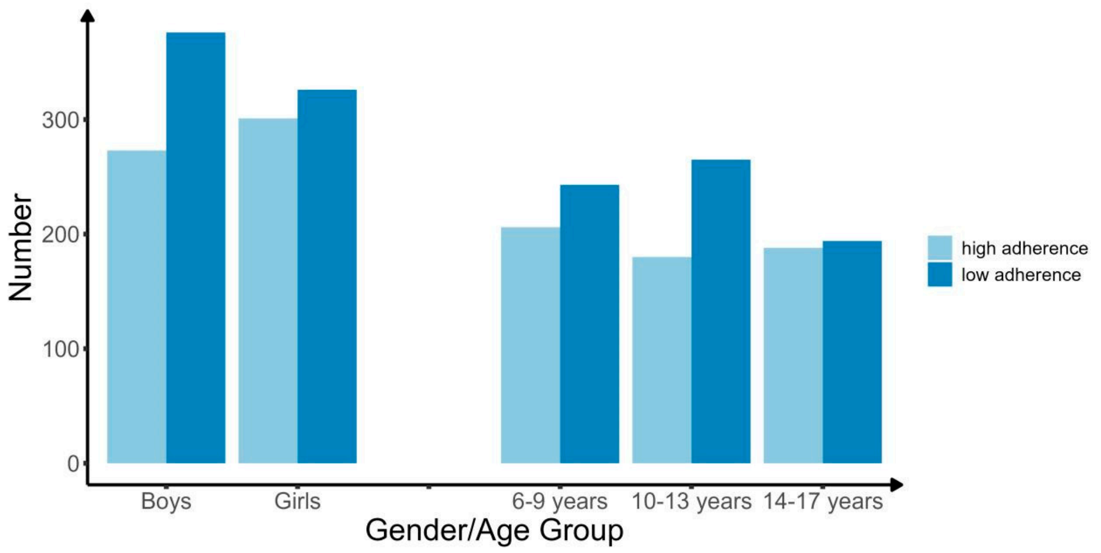

Figure S4. Adherence to a Healthy Lifestyle by Gender and Age Groups.

Table S1. Effect of lifestyle (continues) on thyroid volume and goiter in different iodine status and different age groups.

Thyroid-related indicators	UIC (μg/L)	Age group	Model 1[a]			Model 2[b]		
			β	*SE*	*P*	*β*	*SE*	*P*
**Tvol (ml)**		6-9 y	0.137	0.241	0.572	0.292	0.280	0.301
	**<100**	10-13 y	-0.768	0.298	0.012	-0.739	0.287	0.012
		14-17 y	-0.669	0.594	0.266	-0.495	0.702	0.485
		6-9 y	0.003	0.217	0.989	0.005	0.236	0.984
	**100-199**	10-13 y	-0.326	0.198	0.101	-0.354	0.193	0.069
		14-17 y	-1.062	0.295	0.000	-0.868	0.311	0.006
	**200-299**	6-9 y	-0.142	0.410	0.730	-0.029	0.447	0.948
		10-13 y	-0.179	0.373	0.632	-0.265	0.412	0.522

		14-17 y	-0.638	0.553	0.251	-0.450	0.565	0.428
		6-9 y	0.030	0.142	0.835	0.001	0.148	0.995
	≥300	10-13 y	-1.073	0.367	0.005	-0.795	0.387	0.045
		14-17 y	-0.928	0.353	0.010	-0.770	0.332	0.023
	**UIC (μg/L)**	**Age group**	***OR***	***95%CI***	***P***	***OR***	***95%CI***	***P***
**Goiter**		6-9 y	0.64	0.37-1.12	0.118	0.67	0.35-1.29	0.230
	**<100**	10-13 y	0.76	0.36-1.62	0.482	0.73	0.33-1.612	0.434
		14-17 y	—*	—	—	—	—	—
		6-9 y	0.86	0.55-1.33	0.496	0.90	0.56-1.45	0.668
	**100-199**	10-13 y	0.72	0.42-1.25	0.247	0.59	0.32-1.09	0.089
		14-17 y	0.59	0.25-1.41	0.233	0.67	0.28-1.61	0.374
		6-9 y	0.64	0.34-1.20	0.162	0.66	0.34-1.30	0.230
	**200-299**	10-13 y	0.90	0.44-1.87	0.786	0.64	0.28-1.49	0.303
		14-17 y	—*	—	—	—	—	—
		6-9 y	0.88	0.30-2.64	0.824	0.91	0.32-2.65	0.868
	**≥300**	10-13 y	0.21	0.08-0.55	0.002	0.19	0.06-0.68	0.010
		14-17 y	0.15	0.02-0.99	0.049	0.07	0.00-1.32	0.075

*The dash (—) in the table indicates that the odds ratio (OR), 95% confidence interval (CI), and p-value could not be calculated due to insufficient sample size for reliable statistical analysis.

[a]Model 1 did not adjust for any factors (n=1276).

[b]Model 2 adjusted for gender, age, household income per capita, and the BMI group (n=1184).

Table S2. Effect of lifestyle (count) on thyroid volume and goiter in different iodine status and different age groups.

Thyroid-related indicators	UIC (μg/L)	Age group	*Mean (SD)*	Model 1[a]			Model 2[b]		
				β[#]	*SE*[#]	*P*[#]	*β*[#]	*SE*[#]	*P*
**Tvol (ml)**		6-9 y	3.54 (2.04)	0.149	0.409	0.717	0.224	0.440	0.611
	**<100**	10-13 y	4.83 (2.21)	-1.010	0.492	0.043	-1.055	0.475	0.030
		14-17 y	6.54 (2.74)	-0.362	0.882	0.684	0.512	1.097	0.644
		6-9 y	3.07 (2.12)	0.264	0.371	0.478	0.234	0.401	0.561
	**100-199**	10-13 y	4.78 (2.21)	-0.222	0.352	0.529	-0.380	0.346	0.273
		14-17 y	6.60 (2.87)	-1.855	0.463	0.000	-1.632	0.497	0.001
		6-9 y	3.48 (3.10)	0.137	0.618	0.825	0.333	0.676	0.624
	**200-299**	10-13 y	4.95 (2.71)	-0.342	0.556	0.539	-0.419	0.612	0.495
		14-17 y	6.35 (3.91)	-1.660	0.834	0.049	-1.358	0.869	0.122
		6-9 y	2.94 (1.52)	0.242	0.235	0.305	0.165	0.258	0.524
	**≥300**	10-13 y	4.65 (2.65)	-1.221	0.681	0.077	-0.485	0.716	0.501
		14-17 y	6.42 (2.45)	-1.181	0.484	0.017	-1.012	0.459	0.030
	**UIC (μg/L)**	**Age group**	***N (%)***	***OR*[#]**	***95%CI*[#]**	***P*[#]**	***OR*[#]**	***95%CI*[#]**	***P*[#]**
**Goiter**		6-9 y	28 (26.2%)	0.53	0.21-1.36	0.188	0.61	0.22-1.69	0.340
	**<100**	10-13 y	11 (12.1%)	0.68	0.18-2.55	0.569	0.70	0.17-2.84	0.620
		14-17 y	1(1.9%)	—*	—	—	—	—	—
		6-9 y	35 (16.7%)	1.00	0.47-2.12	0.991	0.94	0.41-2.13	0.878
	**100-199**	10-13 y	22 (9.6%)	0.92	0.35-2.39	0.857	0.70	0.23-2.09	0.525
		14-17 y	10 (5.5%)	0.14	0.02-1.17	0.069	0.17	0.02-1.44	0.105
		6-9 y	21 (14.3%)	0.58	0.21-1.58	0.290	0.62	0.22-1.78	0.374
	**200-299**	10-13 y	16 (12.2%)	0.58	0.18-1.83	0.355	0.41	0.12-1.42	0.161
		14-17 y	4 (3.5%)	2.69	0.27-26.81	0.400	3.49	0.29-41.78	0.324
		6-9 y	7 (7.6%)	1.50	0.24-9.55	0.668	1.50	0.19-11.93	0.704
	**≥300**	10-13 y	10 (11.0%)	0.11	0.01-0.93	0.043	0.23	0.02-2.27	0.209
		14-17 y	3 (2.6%)	—*	—	—	—	—	—

*The dash (—) in the table indicates that the odds ratio (OR), 95% confidence interval (CI), and p-value could not be calculated due to insufficient sample size for reliable statistical analysis.

[#]The coefficients were calculated using the high-risk group as the reference group.

[a]Model 1 did not adjust for any factors (n=1276).

[b]Model 2 adjusted for gender, age, household income per capita, and the BMI group (n=1184).
